# Genome-wide identification and characterization of long non-coding RNAs expressed during sheep fetal and postnatal hair follicle development

**DOI:** 10.1038/s41598-019-44600-w

**Published:** 2019-06-11

**Authors:** Ablat Sulayman, Kechuan Tian, Xixia Huang, Yuezhen Tian, Xinming Xu, Xuefeng Fu, Bingru Zhao, Weiwei Wu, Dan Wang, Aynur Yasin, Hanikezi Tulafu

**Affiliations:** 1 College of Animal Science, Xinjiang Agricultural University, Xinjiang, Republic of China; 2 Institute of Animal Husbandry, Xinjiang Academy of Animal Science, Xinjiang, Republic of China

**Keywords:** Agricultural genetics, Gene expression

## Abstract

Long non-coding RNAs (lncRNAs), >200 nt in length, are transcribed from mammalian genomes. They play important regulatory roles in various biological processes; However, the function and expression profile of lncRNAs involved in the development of hair follicles in the fetus, have been relatively under-explored area. To investigate the specific role of lncRNAs and mRNAs that regulate hair follicle development, we herein performed a comprehensive study on the lncRNA and mRNA expression profiles of sheep at multiple embryonic days (E65, E85, E105, and E135) and six lambs aged one week (D7) and one month (D30) using RNA-seq technology. The number of genes (471 lncRNAs and 12,812 mRNAs) differentially expressed and potential targets of differentially expressed lncRNAs were predicted. Differentially expressed lncRNAs were grouped into 10 clusters based on their expression pattern by K-means clustering. Moreover, Gene Ontology (GO) annotation and Kyoto Encyclopedia of Genes and Genomes (KEGG) pathway analyses showed that some differentially expressed mRNAs, such as *DKK1*, *DSG4*, *FOXE1*, *Hoxc13*, *SFRP1*, *SFRP2*, and *Wnt10A* overlapped with lncRNAs targets, and enriched in important hair follicle developmental pathways, including Wnt, TNF, and MAPK signaling pathways. In addition, 9 differentially expressed lncRNAs and 4 differentially expressed mRNAs were validated using quantitative real-time PCR (qRT-PCR). This study helps enrich the *Ovis* lncRNA databases and provides a comprehensive lncRNA transcriptome profile of fetal and postnatal skin of sheep. Additionally, it provides a foundation for further experiments on the role of lncRNAs in the regulation of hair growth in sheep.

## Introduction

Subo Merino Sheep (SBMS), bred in 2014, are an excellent wool sheep breed in China. This breed is well-known for its good quality wool and high wool yield^1^. Wool, as one of the most valuable products from sheep, is an important material in the textile industry. An improvement in the quality of wool will result in a marked increase in its economic value. Wool follicles, the tissue from which wool is derived, play a vital role in the production of better-quality wool^2^.

Wool is produced through various processes by hair follicles (HFs), which are embedded in the skin of sheep^3^. The HF is a complex accessory organ of the skin that has a unique morphology and structure. HFs consist of epithelial and hypodermal layers, which include the connective tissue sheath, inner root sheath, outer root sheath, hair bulb, and hair shaft^4^. The morphogenesis and growth of HF in sheep has been extensively studied since 1950’s and the developmental processes at the cellular level are reasonably well understood^5,6^.

The HF development directly affects the production and quality of sheep wool. In sheep breeding, the quality and yield of sheep wool can be improved by studying the genes that regulate the development of HF. The structure of the HF in mammals is complex. HF development occurs during fetal skin development, and it is modulated by extra-follicular macro-environmental factors^7,8^. Considering the complexity of the HF, studies on fetal skin have been valuable for fully identifying differentially expressed genes (DEGs) that appear to be developmentally regulated. Although multiple genetic determinants associated with HF formation have been identified, the molecules that are responsible for the independent morphogenesis and development of HF in wool sheep remain elusive.

With the development of molecular, sequencing and bioinformatics analysis technologies, next-generation RNA sequencing technology (RNA-seq) has become a powerful approach that reveals the differential expression profiles underlying phenotypic differences, and deciphers non-annotated transcriptional activity by identifying various novel transcripts (protein-coding and non-coding) and additional alternative splice variants of known annotated transcripts^9–11^. In recent years, RNA-seq has been widely used to identify DEGs and novel transcript units in skin of domestic animals, including sheep^12^, goats^13^, and cattle^14^.

Recent studies looking beyond protein-coding genes have reported that non-coding RNA (ncRNA), such as microRNA (miRNA), natural antisense transcripts (NAT), and long non-coding RNA (lncRNA), can show higher specificity as a bio-marker for various applications (human, sheep, *et al*.) than protein-coding genes^3,15–17^. Among these ncRNAs, a proportion of the lncRNAs encoded by the sheep genome have been annotated but there are still many, including those that are highly tissue specific, that have not been fully characterized^18^. LncRNA is distinct from other subclasses of ncRNA, and represents a novel class of regulatory molecule, arbitrarily defined as transcripts of more than 200 nucleotides (nt) in length that lack specific open reading frames^19^. LncRNAs possess certain characteristics, such as: low number of exons, short sequence length, low sequence conservation, and low expression level^14,20–24^. Recently, lncRNAs have been known to play significant regulatory roles in various biological processes, including X-chromosome inactivation^25,26^, genomic imprinting^27^, developmental processes^28,29^, and disease^30,31^. Currently the identified lncRNAs in lncRNA databases are mainly derived from human, mouse, and bovine^32–35^. Several recent studies on sheep^18,36^, goat^13^, cattle^14,37^, chicken^38^, and pig^29^ have enriched the animal lncRNA databases; however, the role of lncRNAs in regulating the fetal and postnatal HF developmental phases in sheep has not been described at all.

In this study, to understand the function of lncRNAs in skin follicles, we explored the expression profile of lncRNAs in sheep using RNA-seq and quantitative real-time PCR (qRT-PCR). The results demonstrated that lncRNAs may regulate HFs via interacting with their target genes in different signaling pathways. This study of new lncRNAs will form a solid foundation for the further exploration of their roles in regulation of the hair growth cycle and for the wool production of sheep with increased economic value, and will provide a basis for our future work on sheep HF.

## Results

### Overview of sequencing data

In order to identify lncRNAs and mRNAs expressed during the development of HFs in sheep, we constructed 18 cDNA libraries. E65-1, E65-2, and E65-3 as the replicate libraries for the E65 experiment group; E85-1, E85-2, and E85-3 as the replicate libraries for the E85 experiment group; E105-1, E105-2, and E105-3 as the replicate libraries for the E105 experiment group; E135-1, E135-2, and E135-3 as the replicate libraries for the E135 experiment group; D7-1, D7-2, and D7-3 as the replicate libraries for the D7 experiment group; D30-1, D30-2, and D30-3 as the replicate libraries for the D30 experiment group using Subo Merino sheep back-skin samples. Three biological replicates were used for each stage. A total of 1,847,973,012 raw reads were obtained in all 18 libraries. After removing the low-quality reads and adapter fragments, we obtained 1,726,188,919 clean reads, which corresponded to 93.41% of the raw reads from all the libraries. The rRNA content was between 0.03-0.39%; Q20% in all the libraries were above 95%, indicating that clean reads were in good quality. 81–88% of the clean reads in all the libraries were successfully mapped to the reference genome; the average mapping percentage was 81.5%. Out of successfully mapped reads, 82.20% uniquely mapped reads were used in transcript construction. In addition, the sequences of novel transcripts from sheep skin tissue were exist on all chromosomes, except for the mitochondrial genome; the sequences of new transcripts were prevalent on sheep chromosome 1, 2, and 3, which are also the three longest chromosomes.

### Identified lncRNAs

**Figure 1:**
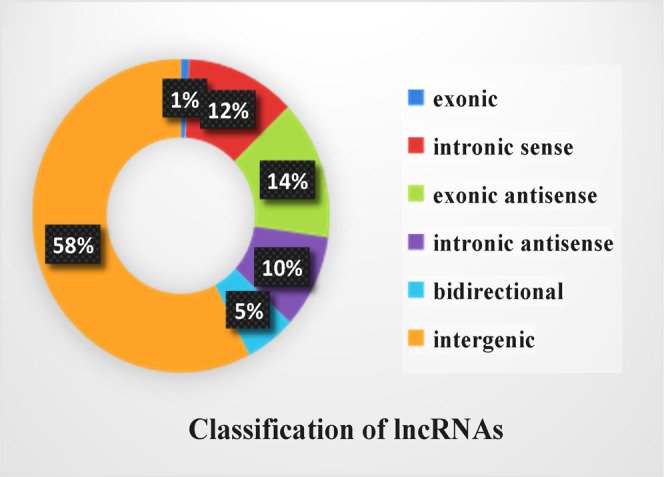
Annotation of genomic context of lncRNAs.

**Figure 2:**
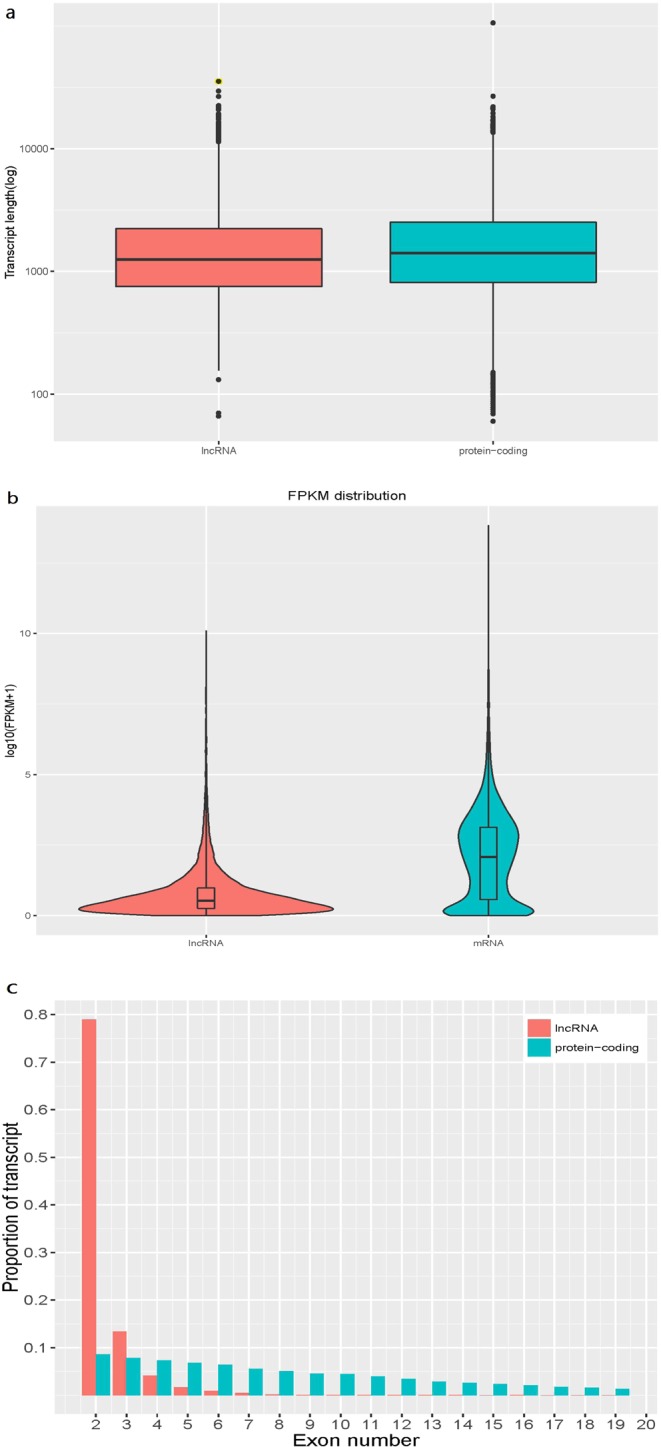
Comparison of genomic features between predicted lncRNAs and mRNAs. (**a**) Distribution of transcript lengths in the lncRNAs and mRNA. (**b**) Expression level indicated by log_10_ (FPKM + 1) in the lncRNAs and mRNAs. (**c**) Distribution of the number of exons in the lncRNAs and mRNAs. The horizontal axis of indicates the number of exons, and the vertical axis represents the proportion of transcript.

After filtering and coding potential evaluation steps, lncRNA transcripts of libraries resulting in 10,193 (1,540 known and 8,653 novel) lncRNAs were considered expressed in skin tissues, respectively, and used in further analyses. In addition, a total of 27,054 mRNAs were identified. To gain insight into the functional role of lncRNAs that are expressed during sheep HF development, we analyzed their genomic context based on their orientation to local protein-coding genes. We identified 95 exonic sense, 1213 intronic sense, 1467 exonic antisense, 988 intronic antisense, 557 bidirectional, and 5873 intergenic lncRNAs (Fig. 1). The exon number, transcript length, and expression level of lncRNAs and mRNAs were calculated and graphed, as shown in Fig. 2a–c. The transcript length (Fig. 2a) and expression level (Fig. 2b) of lncRNAs were significantly shorter than those of mRNAs. 79% of lncRNAs comprised two exons, whereas mRNAs contained a broad range of exon numbers from two to nineteen (Fig. 2c). All in all, lncRNAs were found to be shorter in length, lower expression level, and fewer in exon number than protein-coding genes in skin (P < 0.05)—findings that are in agreement with those of previous studies^9,39^.

### Differentially expressed lncRNAs

**Figure 3:**
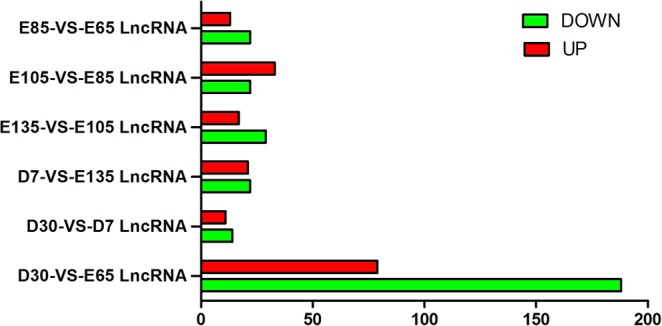
Numbers of up-regulated and down-regulated lncRNAs in sheep skin hair follicle at six developmental stages.

**Figure 4:**
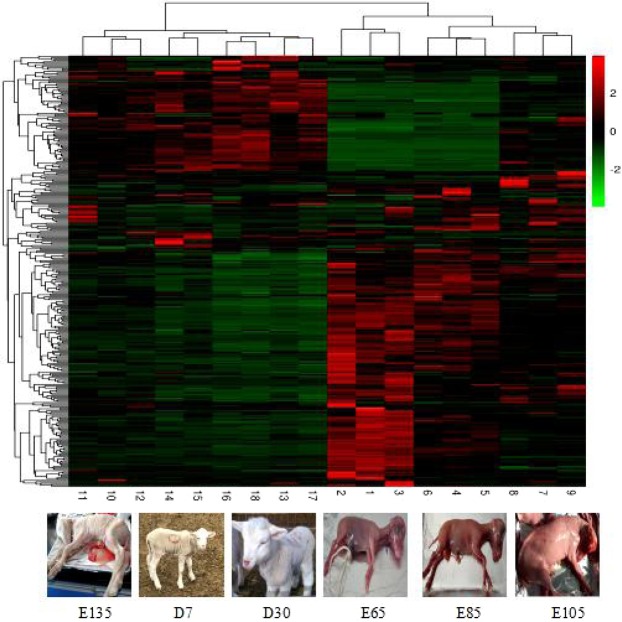
Hierarchical clustering of lncRNAs differentially expressed in fetal and postnatal sheep skin. Data are expressed as FPKM. Red: relatively high expression; Green: relatively low expression. The bar code on the right represents the color scale of the log2 values.

**Figure 5:**
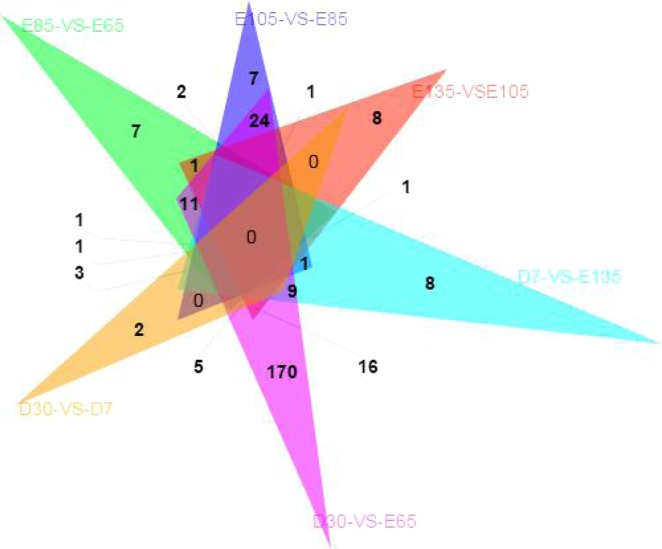
Venn diagram of differentially expressed lncRNAs at the six comparison groups in sheep.

A comparison of lncRNA and mRNAs expression levels were estimated by FPKM (fragments per kilo-base of exon per million fragments mapped) using Cuffdiff. RNA sequencing analyses detected 471 lncRNAs (174 were up-regulated and 297 were down-regulated) (Fig. 3) and 12,812 mRNAs (7,269 were up-regulated and 5,543 were down-regulated) that were differentially expressed in skin during HF development. Additionally, 439 differentially expressed novel lncRNAs were obtained. The number of up-regulated genes of lncRNA was greater than that of down-regulated genes at the E105-VS-E85 comparison group, while the number of down-regulated genes was greater than that of the up-regulated genes in skin at other comparison groups. The expression patterns of differentially expressed lncRNAs were measured by systematic cluster analysis, to explore the similarities and to compare the relationships between the different libraries. The replicates for each developmental stage clustered together, and E65, E85, and E105 formed one group and E135, D7, and D30 formed another group Data are expressed as FPKM. Red: relatively high expression; Green: relatively low expression. The bar code on the right represents the color scale of the log2 values (Fig. 4). To further analyze the interactions among the differentially expressed lncRNAs, we constructed a Venn diagram using the 35, 55, 46, 43, 267, and 27 lncRNAs that were differentially expressed in E85-VS-E65, E105-VS-E85, E135-VS-E105, D7-VS-E135, D30-VS-E65, and D30-VS-D7, respectively (Fig. 5). We did not detect any co-expressed differentially expressed lncRNA in all six developmental stages, but we identified 7, 7, 8, 8, 170, and 2, respectively, stage-specific differentially expressed lncRNAs. We then compared the expression level of these differentially expressed lncRNAs between the samples for every two HF stages. Even though, the level of expression of most of these lncRNAs was equivalent, there were differences in the level of expression between the two stages for some lncRNAs.

### Target gene prediction of lncRNAs

To understand the function of the differentially expressed lncRNAs in different HF developmental stages, we predicted the target genes of cis- and trans-acting lncRNAs. A total of 3,685 potential target genes were identified for 5,852 cis-acting lncRNAs, while 1,722 potential target genes were identified for 4,117 trans-acting lncRNAs. These 3,685 and 1,722 candidate target genes were subjected to GO and pathway analyses.

### GO enrichment and KEGG pathway analysis of lncRNA targets and mRNAs

Functional analysis showed that cis and trans target genes of lncRNAs were enriched in 5,509 GO terms. GO terms of lncRNA potential targets were highly enriched for several processes, such as organic substance metabolic, regulation of stimulus, and cellular metabolic process. Importantly, 13, 5, 12, and 7 predicted lncRNA targets overlapped with the differentially expressed mRNAs, and predicted to function in HF development and morphogenesis, epidermis development and morphogenesis, skin development and morphogenesis, and cell differentiation and migration, respectively (see Table 1).

**Table 1:** Summary of differentially expressed lncRNA targets and mRNAs involved in hair follicle and skin development.

Hair follicle development, morphogenesis, and hair cycle process	lncRNA targets	**DKK1 KRT25 HOXC13 FOXE1** SOS1 **DSG4 LHX2** CDH3 MYSM1 ERCC2 CD109 **FZD3 ALX4 LGR5** RELA **CELSR1 WNT10A** LDB1 **LRP4 FGF10** FST
	mRNA	**DKK1 KRT25 HOXC13 FOXN1** LGR4 ACVR1B SOX9 SOSTDC1 **DSG4** TP63 LAMA5 NOTCH1 TGFB2 LHX2 INHBA ZDHHC21 DNASE1L2 KRT71 **LHX2** TMEM79 **FZD3** FZD6 **ALX4 LGR5** KRT71 TNF-alpha **CELSR1** MSX2 EGFR KRT17 **WNT10A** KRT27 FOXN1 SMO APCDD1 **LRP4** SNAI1 **FGF10**
Epidermis development and morphogenesis	lncRNA targets	WNT16 **DKK1 KRT25 DSG4** SOS1 **FOXE1 HOXC13** OVOL1
	mRNA	FOXN1 INHBA TGFB2 LRP4 NOTCH1 KDF1 WNT10A LAMA5 **DKK1** EDAR CELSR1 LGR5 TP63 ALX4 **KRT25** DLL1 SOSTDC1 SOX9 **DSG4** LHX2 ACVR1B LGR4 VDR TMEM79 ALOX15B SFN MSX2 CYP27B1 H2AFY2 **FOXE1** KRT36 MAFF KRT84 HES5 DNASE1L2 KRT27 CST6 **HOXC13**
Skin development and morphogenesis	lncRNA targets	**WNT16 CLDN1 COL5A1 DKK1 DHCR24 KRT25 ALOXE3 DSG4 COL5A2** SOS1 PSEN1 **JUP** OVOL1 **FOXE1 HOXC13**
	mRNA	FOXN1 TGFB2 INHBA KDF1 **WNT16** COL1A1 ALOX12B TFAP2B EDAR **CLDN1** VDR LAMA5 **COL5A1** DLL1 LGR5 **DKK1 DHCR24** GRHL2 **KRT25** ALX4 SOX9 SOSTDC1 FA2H PTCH2 ACVR1B LHX2 ASPRV1 CSTA **ALOXE3** COL1A2 ITGA3 CYP26B1 LRP4 NOTCH1 WNT10A TGM3 ACER1 CELSR1 ITGB4 ABCA12 TP63 ATP8A2 WNT5A LGR4 KRT1 FRAS1 TCF15 KRT27 **DSG4 COL5A2 JUP FOXE1** DNASE1L2 SFN **HOXC13** KRT71
Cell differentiation, death, and migration	lncRNA targets	**SFRP1** PPP1R16B CDH5 IL1B ENG ALMS1 PSMB8 SFRP2 **WNT5B** DKKL1 TRAF6 PSEN1 TMEM176A IRF4 LHX8 FGB PM20D1 **WNT16** PAX8 PTTG1IP NLE1 AURKB **DPEP1** CIB1 TAF9B TEX11 FGG CDK1 PDK4 **TDGF1** IDO1 MAEL **CLEC5A** HSP90B1 RBM10 **PTK2B** IGF1 AIFM2
	mRNA	NOTCH1 MYCN **SFRP1 WNT5B** DLL1 **WNT16** HES5 PDZD7 MFSD2A ITGA8 TRIM62 TH FOXB1 TGFB2 LGI1 **DPEP1** MYRF PLA2G3 PTCH2 LAMA5 FZD1 EDN1 **TDGF1** NOTCH1 **CLEC5A** LTBP3 TGM3 PRDM1 CTGF UNC5C KLF5 GRIP1 ISG15 BMPER BMP2 TNR **PTK2B** TFAP2 PTCH1
WNT signaling pathway	lncRNA targets	**WNT16 SFRP1 FZD4 SFRP2 DKK1** FBXW11 PSEN1 **HOXC13** CSNK1A1 **DAAM1**
	mRNA	WIF1 **WNT16 SFRP1** MMP7 WNT10A FZD1 SFRP4 WNT3 WNT5A SFRP5 SERPINF1 CAMK2A BAMBI **FZD4** FZD5 CSNK1E **SFRP2** NOTUM PLCB4 WNT2 WNT2B FZD3 VANGL1 **DKK1** NFATC4 DAAM2 FZD6 PRICKLE1 WNT8B PPARD **HOXC13** PRKACB FRAT2 FOSL1 **DAAM1**
TNF signaling pathway	lncRNA targets	IL1B IL18R1 DNM1L **JUNB**
	mRNA	PIK3R5 CREB3L4 SELE MAP2K6 EDN1 NOD2 ICAM-1 PIK3CB IL15 FOS CXCL10 PIK3R3 **JUNB** MLKL MAP3K5 MMP14 MMP9 MMP3 TNF-alpha
MAPK signaling pathway	lncRNA targets	IL1B MAPKAPK5 TRAF6 NTRK2 **PDGFRA PLA2G4D** SOS1
	mRNA	IL1R1 MAP2K6 DUSP4 IL1A TGFB2 CACNA2D3 PLA2G4F CACNA1D FGF19 CACNB1 GADD45B FOS BDNF DUSP2 **PDGFRA** HSPA1B RASGRP2 IL1R2 PTPRR DUSP1 CACNG6 MAP3K6 HSPA2 MAPKAPK3 HSPA6 MAP3K5 **PLA2G4D** PLA2G4E MRAS FLNC FGF9 TGFB3 MEF2C FGFR4
Hedgehog signaling pathway	lncRNA targets	FBXW11 CSNK1A1 **CDON**
	mRNA	HHIP GLI1 GLI2 CSNK1E LRP2 **CDON** GPR161 ARRB1 SMO PRKACB CUL3

Genes marked in bold indicate the potential lncRNA targets that overlapped with mRNAs in the important hair follicle developmental stages.

Based on the KEGG pathway database, pathway analysis was performed to predict the significantly enriched pathways involving putative target genes of differentially expressed lncRNAs. Pathway analysis showed that 268 pathways involving the target genes of differentially expressed lncRNAs were enriched; some of these pathways are known to play an important role in the regulation of HF development, including Wnt, TNF, MAPK, and Hedgehog which shared 7, 1, 2, and 1 genes with lncRNA targets, respectively (see Table 1). To characterize the GO terms and pathways, we listed the top 30 significantly enriched GO terms and pathways (*P* < 0.05) in six comparative groups.

**Figure 6:**
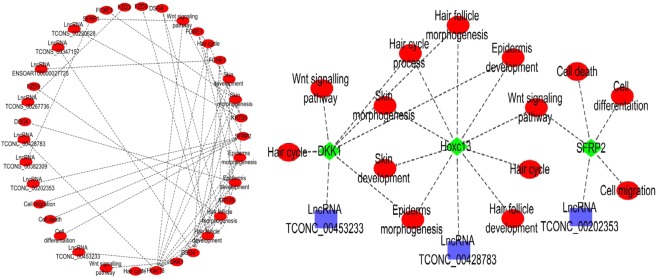
GO and KEGG network relationships of hair follicle development related LncRNA target genes.

It is worth mentioning that SFRP2^40^, DKK1^41^, and Hoxc13^42^ obtained from the GO enrichment and KEGG pathway analysis of lncRNA targets and mRNAs have been proved to be key genes related to HF development, and in this experiment, these three genes are enriched in GO items and Wnt signaling pathway related to HF development. SFRP2, DKK1, and Hoxc13 are the predicted target genes of the differentially expressed lncRNAs such as TCONS_00202353, TCONS_00453233, and TCONS_00428783, which may regulate the development of HF (Fig. 6).

### K-means cluster analysis of lncRNAs

**Figure 7:**
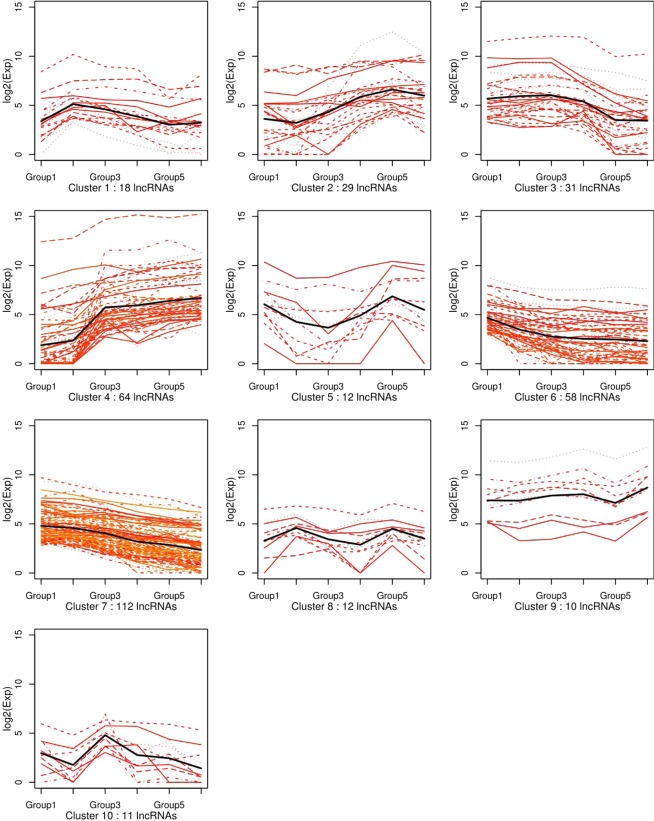
K-means clustering analysis of differentially expressed lncRNAs among six comparison groups. Each box is a differential lncRNA cluster, each line in the box represents a expression trend of differential lncRNAs, a black bold lines is the average trend of all differential lncRNAs in the box.

To identify the expression pattern of differentially expressed lncRNAs during six HF developmental stages, we performed k-means clustering of all differential lncRNAs. LncRNAs were classified into 10 clusters based on their group mean and changes with respect to other compartments. Each box is a differential lncRNA cluster, each line in the box represents a expression trend of differential lncRNAs, a black bold lines is the average trend of all differential lncRNAs in the box (Fig. 7). These clusters can be further assigned to four main groups of different dynamic patterns. The first pattern group, comprising clusters 2 and 4 included 29 and 64 differentially expressed lncRNAs, represents lncRNAs whose expression level gradually increased from that of lncRNAs in Group 2. The second pattern group, comprising of clusters 1, 3, 6, and 7 included 18, 31, 58, and 112 differentially expressed lncRNAs respectively, represents lncRNAs that showed an overall decreasing trend. The third pattern group, consisting of clusters 5, 8, and 10 included 12, 12, and 11 differentially expressed lncRNAs respectively, represents lncRNAs that were up- and down-regulated continuously in all stages. The expression level of 10 differentially expressed lncRNAs in cluster 9, which is the last pattern group, remained stable at the entire stages.

### Validation of the sequencing data by qRT-PCR

**Figure 8:**
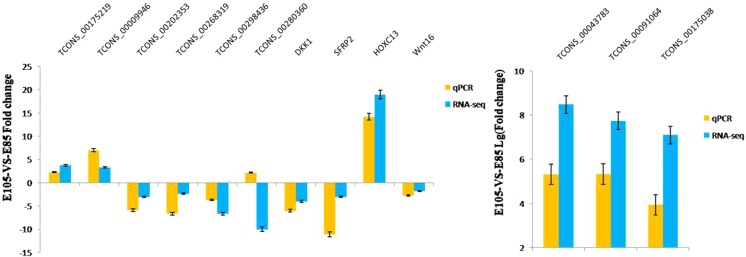
The expression level of differentially expressed lncRNAs validated by qRT-PCR.

To validate the sequencing data, we investigated the expression pattern of 9 randomly selected lncRNAs and 4 mRNAs, which were identified to be differentially expressed during two HF developmental stages (E85 and E105; because these two groups are the important stages for the primary HF development and secondary HF formation)^43^ using qRT-PCR. As shown in Fig. 8, all of the selected lncRNAs and mRNAs were detected in skin tissues, and of these, 12 showed differential expression at two different time points. In addition, TCONS_00280360 did not show differential expression, and this result was inconsistent with that of RNA-seq. Collectively, the sequencing results were reliable.

## Discussion

Several lncRNAs relevant to skin biology have been reported, such as ANCR and SPRY4-IT1^44^, the detailed functional roles of lncRNAs in hair and wool follicle development are based on limited reports and, thus, largely unknown. A recent study detected 15 differentially expressed lncRNAs during secondary wool follicle induction in super-fine wool sheep, which possesses no primary wool follicles due to selective breeding^12^. In recent years, increasing evidence has demonstrated that lncRNAs play important roles in the development of various tissues, such as brain^45^, liver^46,47^, heart^48^, testis^49–51^, ovary^52^, kidney^53^, uterus^54^, muscle^55–57^, mammary gland^58^, and inner ear^59^. However, very few studies have been conducted on the potential role of lncRNAs in mammalian skin HF development^3,36^. Regarding sheep skin tissues, investigations into the molecular mechanisms of HF development have focused more on miRNAs^60–62^ than on lncRNAs, and therefore, the role of lncRNAs in sheep HF development has not been fully illustrated.

In the present study, we systematically identified the lncRNAs and mRNAs involved in sheep (*Ovis aries*) skin during different HF development across four fetal and two postnatal stages, using RNA-sequencing technology. We subsequently characterized putative lncRNAs to elucidate their diverse features in order to provide an insight into the uncharacterized sheep genome regions and their relationship with sheep HF development. To the best of our knowledge, this is the first study to systematically identify lncRNAs from RNA-seq data of sheep HF development during fetal and postnatal stages. In addition, we identified a total of 10,193 and 27,054 expressed lncRNAs and mRNAs in different sheep HF developmental stages; in which, many lncRNAs that were not reported in previous data were identified.

Unlike protein-coding genes or miRNAs, the function of lncRNAs could not be inferred from previous sequencing or structural studies performed by us and others^63,64^. Therefore, we analyzed the genomic context of identified lncRNAs and classified them based on their genomic relationship with protein-coding genes as exonic sense, intronic sense, exonic antisense, intronic antisense, bidirectional, and intergenic. We found that all six categories of lncRNAs could be examined. LcRNAs identified in sheep skin shared many of the features of other mammalian counterparts: shorter length, lower expression level, and fewer exon number than those of protein-coding genes^37,62^. These clues further indicate that mammalian lncRNAs share some specific characteristics, and provide a reference for studying lncRNAs in other species.

A total of 471 differentially expressed lncRNA genes were identified in fetal and postnatal skin tissues. We also performed clustering analysis on 18 samples showing differential expression of lncRNAs at different HF developmental stages. The results indicated that the early stages of HF development (E65, E85, and E105) are clustered together, while the later stages of HF development (E135, D7, and D30) are clustered together. The above findings suggest that the changes in the expression of these lncRNAs in early stages of HF development are much more dramatic than those in later stages. To further survey the interaction of differentially expressed lncRNAs genes, we constructed a Venn diagram using lncRNAs among all the developmental stages. As a result, 7, 7, 8, 8, 170, and 2 stage-specific differentially expressed lncRNA genes were identified out of the 35, 55, 46, 43, 267, and 27 lncRNA genes, respectively. These results suggested that they may have an important role in HF development.

Most evidence suggests that the expression of lncRNA-coding genes can regulate and have a high correlation with the expression of neighboring mRNA-coding genes^63,64^. Based on this, we searched for coding genes 10-kb upstream and downstream of lncRNA-coding genes as target genes to predict the function of the cis-acting lncRNAs. We also searched for mRNA-coding genes using co-expression prediction to predict function of trans-acting lncRNAs. Consequently, we found that many target genes of differentially expressed lncRNAs were also differentially expressed. This suggested that lncRNAs may function through cis/trans-regulation of target genes, and this may play a critical role in the development of sheep skin HFs.

We identified 471 differentially expressed lncRNAs genes and predicted their potential targets which partially overlapped with differentially expressed mRNAs and were enriched in HF developmental GO terms. Pathway analysis showed that 268 pathways involving the target genes of differentially expressed lncRNAs were enriched; some of these pathways are known to play an important role in the regulation of HF development, such as WNT^65^, TNF^66^, and MAPK signaling pathways^67^ (Table 1). Interestingly, we found potential target genes, including *DKK1*, *Hoxc13*, *DSG4*, *Wnt10A*, *FOXE1*, *SFRP1*, and *SFRP2* were overlapped with mRNAs, and enriched in the HF developmental pathways mentioned above, and were found to play important roles in HF development and morphogenesis^68–74^. Recently, many studies claimed that lncRNAs could promote or inhibit the expression of target genes by elevating or reducing the activity of pathways^75–77^. It was inferred that the above-mentioned lncRNAs, and their target genes could regulate the HF developmental process via regulating various pathways.

K-means cluster analysis indicated that differentially expressed lncRNAs in the first pattern group had a positive correlation, and play important roles in the development of HF, while the differentially expressed lncRNAs in the second pattern group had a negative correlation, and lack of involvement in HF development. In addition, differentially expressed lncRNAs in the third pattern group had a complex regulatory role on the development of HF. Furthermore, differentially expressed lncRNAs in the last pattern group might be involved in the HF development at the postnatal stage. To validate the sequencing data, we randomly selected 9 different lncRNAs and 4 mRNAs for qRT-PCR analyses. Except TCONS_00280360, the qRT-PCR results of other selected lncRNAs and mRNAs are consistent with the sequencing data. RNA-seq is used for large-scale screening, which reflects the overall trend of gene expression in the sample, but does not guarantee that the trend of each gene is consistent with qRT-PCR. Generally speaking, the qRT-PCR result is more accurate. RNA-Seq and qRT-PCR have different experimental conditions and may get different results. Collectively, the sequencing results were reliable.

In conclusion, we systematically identified the lncRNAs and mRNAs involved in sheep (Ovis aries) skin during different HF development across four fetal and two postnatal stages using RNA-sequencing technology. A total of 471 differentially expressed lncRNAs and 12,812 differentially expressed mRNAs were identified from all the lncRNA libraries. GO and KEGG function enrichment analysis indicated that some target genes of these lncRNAs as DKK1, Hoxc13, DSG4, Wnt10A, FOXE1, SFRP1, and SFRP2 were overlapped with mRNAs, and involved in HF developmental GO terms and pathways. The dynamic expression profile and analysis of certain features of these genes indicated that lncRNAs may play important roles in HF development of sheep. These results will serve as a very useful resource for genetic information that may improve our understanding of the mechanism of action lncRNAs involved in skin HF development.

## Materials and Methods

### Ethics statement

The methods were performed in accordance with the guidelines of the good experimental practices adopted by the Institute of Animal Husbandry. All experimental protocols were approved by the Institute of Animal Husbandry of the Xinjiang Academy of Animal Science.

### Animal selection and skin tissue preparation

The Subo Merino sheep, a breed of sheep in China, is famous for its excellent quality and yield of wool, and high survival rate. The tested individuals were selected from the Subo Merino sheep herd located at Xinjiang Kechuang animal husbandry breeding center, Urumqi county (Latitude 43°01′08″–44°06′11″N, Longitude 86°37′56″–88°58′22″E), Xinjiang Province, China. 40 Healthy Subo Merino ewes (2~3 years old), which were selected from the same flock and reared using the same management practices, were artificially inseminated with fresh sperm from a single ram. The day of insemination was designated as embryonic day 0 (E0). Three embryos and three lambs (three samples at each stage) were collected. Skin samples (~2 cm^2^ for each individual) were collected from the right mid-side of embryos at four different embryonic days (E65, E85, E105, and E135) and from lambs at two postnatal days (D7 and D30). The samples were cut into small pieces, quickly placed into liquid nitrogen, and subsequently stored at −80 °C for RNA-seq and qRT-PCR analyses.

### Total RNA isolation, library construction, and sequencing

Total RNA was isolated from the tissues using TRIzol reagent (Invitrogen, Carlsbad, CA, USA). RNA quality was verified using a 2100 Bioanalyzer RNA Nano Chip (Agilent technologies, Santa Clara, CA, USA), and the total RNA content was measured using a Nanodrop ND-2000 Spectrophotometer (NanoDrop Technologies, Wilmington, DE, USA). Only the samples with RNA Integrity Number (RIN) scores >8 were used for sequencing. 18 cDNA libraries were constructed using the NEBNext® Ultra^TM^ Directional RNA Library Prep Kit for Illumina (NEB, USA) according to the manufacturer’s instructions, after the removal of rRNA using Epicentre Ribo-Zero rRNA Removal Kit (Epicentre, USA). Purified libraries were quantified using a Qubit® 2.0 Fluorometer (Life Technologies, USA), and validated using an Agilent 2100 bioanalyzer (Agilent Technologies, USA) to confirm the insert size and calculate the molar concentration. The clusters were generated on a cBot Cluster Generation System using the TruSeq PE Cluster Kit v3-cBot-HS (Illumina), and they were subsequently sequenced using the Illumina HiSeq 2000 (Illumina, USA) to generate 150 bp paired-end reads.

### Quality control and transcriptome assembly

Sequencing-received raw image data were transformed by base calling into sequence data, which was called raw data. The raw data were subjected to quality control using FastQC (v0.11.4) (http://www.bioinformatics.babraham.ac.uk/projects/fastqc/). Raw reads of all 18 samples were pre-processed using in-house Perl scripts developed by the Novogene Bioinformatics Institute (Beijing, China). In this step, clean data were obtained after filtering out reads with adapters and poly-N > 10%, and low quality reads (>50% of bases whose Phred quality scores were <5%) from the raw data. At the same time, the Q20 (the 1% base error rate) and GC (the percent of guanine and cytosine) content of the clean data were calculated.

All subsequent analyses were based on the high-quality data. The clean reads were then mapped to the sheep reference genome (ensembl Oar v3.1) using TopHat (v2.0.9)^10^. Reference genome and gene model annotation files were downloaded from Ensembl browser (ftp://ftp.ensembl.org/pub/release-85/fasta/Ovis_aries/dna/Ovis_aries.Oar_v3.1.dna.toplevel.fa). The mapped reads from each sample were assembled by both Scripture (beta2)^78^ and Cufflinks (v2.2.1)^9^ in a reference-based approach. Scripture was run with default parameters. Cufflinks was run with ‘min-frags-per-transfrag = 0’ and ‘—library-type fr-firststrand’, and other parameters were set as default. Read counts for different gene and other known transcripts (misc_RNA, pseudogene, rRNA, tRNA, and others) were extracted by HTSeq (v0.6.1)^79^ with default parameters and allowing one mismatch. To monitor mapping events on both strands, both sense and antisense sequences were included^80^.

### Identification of lncRNAs

**Figure 9:**
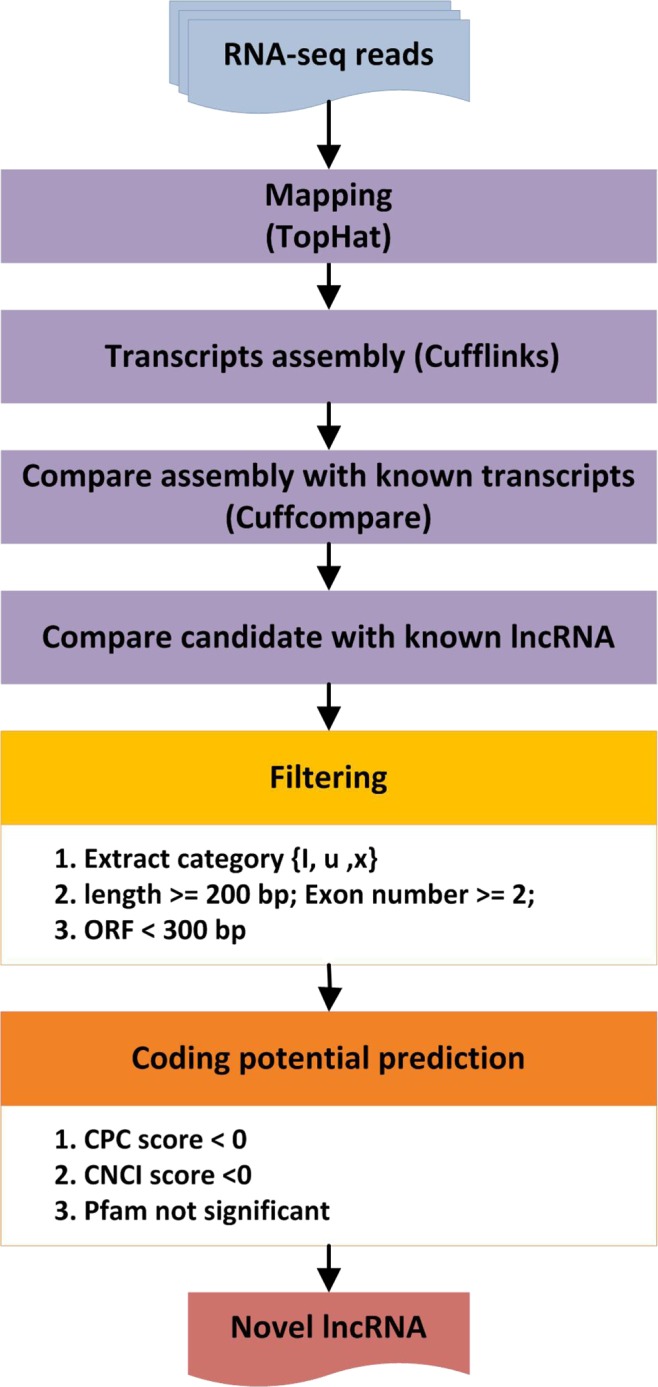
The workflow of novel lncRNA prediction.

Putative lncRNAs were defined as novel transcripts if they satisfied the following criteria: (1) Transcripts with single-exon and less than 200 bp in length were removed first; (2) All transcripts encoding complete open reading frames (ORFs) of more than 300 bp were excluded from our dataset; (3) Transcripts that did not belong to any other type of RNAs (rRNA, tRNA, snRNA, snoRNA, pre-miRNA, and those encoded by known pseudogenes) were excluded; (4) Among the different classes of class_code (http://cufflinks.cbcb.umd.edu/manual.html#class_codes), only transcripts annotated as “u”, “i”, and “x” were retained, which represent potential novel intergenic, intronic and anti-sense lncRNAs, respectively; (5) In order to identify known lncRNAs, BLASTN tool (v2.2.26+, e-value = 1e − 10) was used to align lncRNA candidates to ALDB (A Domestic Animal Long Noncoding RNA Database)^81^, a database with a focus on the domestic-animal lncRNAs, with the settings of identity = 100%, mismatch = 0, E-value; (6) Three coding potential analyses software, Coding-Non-Coding-Index (CNCI) (v2)^82^ (score < 0), Coding Potential Calculator (CPC) (0.9-r2)^83^ (score < 0), and Pfam-scan (v30.0)^84^ (E-value < 0.001) were used to discover the candidate lncRNAs. Transcripts predicted with coding potential by any of the three tools earlier described were filtered out, and those without coding potential were retained. Then, we selected those shared by three tools as the final candidate lncRNAs and use for the further analysis. For mRNA identification, uniquely mapped and properly paired reads were used in transcript construction with Cufflinks (v2.2.1)^85^ for mRNA identification. Constructed transcripts were compared with Ensembl *Ovis* aries gene annotation to identify expressed mRNAs using Cuffcompare^85^. The detailed workflow of novel lncRNA prediction is presented in Fig. 9.

### Differential expression analysis and hierarchical clustering of lncRNAs

Cuffdiff (v2.1.1) was used to calculate fragments per kb for a million reads (FPKM) of both lncRNAs and mRNAs in each sample^10^, based on the TopHat BAM files and the reference GTF file. These counts were used in analyses of differential gene expression using the edgeR v3.16.0 software^86^. LncRNAs and coding genes were differentially expressed between any two comparison groups of samples which representing a specific developmental stages were identified using edgeR^86^. For biological replicates, lncRNAs and mRNAs with a *P*-adjust ≤ 0.05 and an absolute value of the log2(fold change) ≥2 were assigned as being differentially expressed between different comparison groups^13^. The expression patterns of differentially expressed lncRNAs were measured by systematic cluster analysis, to explore the similarities and to compare the relationships between the different libraries. Scatter plots were used to demonstrate lncRNAs showing differential expression between every two HF developmental stages. Furthermore, the differentially expressed lncRNAs were subjected to K-means clustering using the Euclidean distance method associated with complete linkage on the BMKCloud platform (https://www.biocloud.net/).

### lncRNA genomic context analysis

We determined the genomic context of lncRNAs in relation to protein-coding genes according to a protocol that was updated for this study^87^. Briefly, intronic sense and antisense lncRNAs were defined, and the sequence of each corresponding transcript was mapped to the positive and opposite strand, respectively, of an intron of a protein-coding gene. Exonic sense and antisense lncRNAs were defined, and the sequence of each corresponding transcript was mapped to the positive and opposite strand, respectively, of a RefSeq-annotated exon [includes 5′-untranslated region (UTR), coding exon, and 3′-UTR]. Bidirectional lncRNAs were defined, and the sequence of each corresponding transcript was mapped head-head to protein-coding gene at a distance of <1000 bp. Intergenic lncRNAs were defined, and the sequence of each corresponding transcript was mapped within an intergenic region, without the presence of any overlapping or bidirectionally coded sequences for transcripts nearby.

### Target gene prediction

Since lncRNAs can cis target the neighboring genes^88,89^, we searched coding genes 10-Kb upstream and downstream of all identified lncRNAs and analyzed their functional roles. Firstly, we computed Pearson’s correlation coefficients between each pair of lncRNA–protein coding genes. The protein coding genes having significant correlations with lncRNAs at p.BH < 0.05 were considered potential cis target genes for those lncRNAs. The trans-acting lncRNAs influence the expression of other genes. For the trans target gene prediction, the blast ratio (e < 1E-5) between lncRNAs and protein coding genes was calculated. RNAplex software was then used to select trans-acting target genes^90^.

### GO and KEGG pathway enrichment analysis

To improve our understanding of the functions of the lncRNAs and mRNAs, mRNAs and potential cis and trans target genes of lncRNAs were mapped to GO terms in the Gene Ontology (GO) database, and the gene numbers for each GO term were calculated using the GOseq R package (v1.18.0)^91^. In addition, Kyoto Encyclopedia of Genes and Genomes (KEGG) enrichment analysis of target genes and mRNAs was performed using the KOBAS software (v2.0)^92^ using a hypergeometric test. GO terms and KEGG pathways with P < 0.05 were considered significantly enriched. The calculating formula is:

$$P=1-\sum _{i=0}^{m-1}\frac{(\begin{array}{c}M\\ i\end{array})(\begin{array}{c}N-M\\ n-i\end{array})}{(\begin{array}{c}N\\ n\end{array})}$$

where ʻNʼ is the number of all lncRNAs with GO or KEGG annotation; ʻnʼ is the number of lncRNAs in ʻNʼ; ʻMʼ is the number of genes that are annotated to the certain GO terms or specific pathways; and ʻmʼ is the number of DEGs that are annotated to the certain GO terms or specific pathways in ʻMʼ.

### qRT-PCR

Several differentially expressed mRNAs and lncRNAs involved in HF development were selected and confirmed by qRT-PCR with Ovis-actin used as an internal reference. The primers for qRT-PCR are listed in Supporting Information 1. Total cDNA was synthesized using ReverTra Ace qPCR kit (TOYOBO). qPCR was performed on the 7900 HT Sequence Detection System (ABI, USA) using ABI Power SYBR Green PCR Master Mix (ABI, USA). Quantitative PCR was performed under the following conditions: denaturation at 98 °C for 5 min, followed by 40 cycles of amplification and quantification (95 °C for 15 s, and 60 °C for 1 min). Melting curves were analyzed after amplification. Except for the cDNA conversions, the other reactions were run in triplicate for each sample. The delta-delta-Ct method was used to analyze the expression level^93^.

## Supplementary information

Supplementary information

Dataset 1

Dataset 2

Dataset 3

Dataset 4

Dataset 5

Dataset 6

Dataset 7

Dataset 8

Dataset 9

Dataset 10

Dataset 11

Dataset 12
