# Supplementary material for: Genome-wide identification and characterization of long non-coding RNAs expressed during sheep fetal and postnatal hair follicle development
Source: Sci Rep. 2019 Jun 11;9:8501. doi: 10.1038/s41598-019-44600-w (PMC6559957; doi:10.1038/s41598-019-44600-w)
Supplement: Supplementary file 1 — Supplementary information [file 41598_2019_44600_MOESM1_ESM.docx]

**Genome-wide identification and characterization of long non-coding RNAs expressed during sheep fetal and postnatal hair follicle development**

Ablat Sulayman^1,2^, Kechuan Tian^2,^*, Xixia Huang^1,^*, Yuezhen Tian^2^, Xinming Xu^2^, Xuefeng Fu^2^, Bingru Zhao^1^, Weiwei Wu^2^, Dan Wang^1^, Aynur Yasin^2^ and Hanikezi Tulafu^2^

^1^ College of Animal Science, Xinjiang Agricultural University

^2^ Institute of Animal Husbandry, Xinjiang Academy of Animal Science

***Correspondence:** [tiankechuan@163.com](mailto:tiankechuan@163.com); [au-huangxixia@163.com](mailto:au-huangxixia@163.com)

**Supplementary Tables**

**Supplementary Table S1. Summary of read numbers based on the RNA-seq from Subo Merino sheep HF development.**

| **Samples** | **Raw reads** | **Clean reads** | **Clean ratio** | **rRNA ratio** | **Q20（%）** | **GC（%）** |
| --- | --- | --- | --- | --- | --- | --- |
| E65-1 | 74658372 | 70109427 | 93.91% | 0.05% | 97.16% | 47 |
| E65-2 | 90071904 | 85079501 | 94.46% | 0.04% | 96.98% | 47 |
| E65-3 | 81756998 | 76533092 | 93.61% | 0.03% | 96.91% | 46 |
| E85-1 | 98571166 | 91875538 | 93.21% | 0.03% | 96.83% | 46 |
| E85-2 | 115594538 | 106206329 | 91.88% | 0.05% | 97.05% | 46 |
| E85-3 | 108469680 | 102105039 | 94.13% | 0.06% | 96.93% | 47 |
| E105-1 | 77264650 | 71835595 | 92.97% | 0.06% | 96.12% | 48 |
| E105-2 | 77462114 | 72259875 | 93.28% | 0.08% | 96.05% | 48 |
| E105-3 | 76879058 | 71062871 | 92.43% | 0.25% | 95.82% | 48 |
| E135-1 | 77981432 | 72051361 | 92.40% | 0.21% | 95.95% | 48 |
| E135-2 | 117438066 | 109978573 | 93.65% | 0.30% | 96.84% | 49 |
| E135-3 | 97065620 | 91061815 | 93.81% | 0.26% | 96.95% | 48 |
| D7-1 | 141926862 | 134529942 | 94.79% | 0.20% | 97.20% | 47 |
| D7-2 | 219640918 | 207587692 | 94.51% | 0.17% | 97.06% | 48 |
| D7-3 | 73091410 | 68433629 | 93.63% | 0.15% | 95.74% | 47 |
| D30-1 | 123422552 | 114514723 | 92.78% | 0.16% | 96.54% | 47 |
| D30-2 | 105969718 | 96414815 | 90.98% | 0.39% | 96.73% | 49 |
| D30-3 | 90707954 | 84549102 | 93.21% | 0.29% | 96.80% | 48 |

Clean ratio=(Clean reads/Raw reads)%; rRNA ratio=1 - [(Clean reads - rRNA trimmed)/ Clean reads]%

**Supplementary Table S2. Summary of clean reads mapping to the *Ovis_aries*_v3.1 reference genome sequence.**

| **Samples** | **Cleaned total reads** | **Mapped reads** | **Mapped Pair**  **Reads** | **Mapped Unique**  **reads** | **Mapped Multi**  **reads** | **Mapping ratio** |
| --- | --- | --- | --- | --- | --- | --- |
| E65-1 | 70073894 | 61175625 | 55960318 | 59069253 | 2106372 | 87.30% |
| E65-2 | 85043435 | 74253627 | 67398024 | 72054965 | 2198662 | 87.31% |
| E65-3 | 76507605 | 67410760 | 61352066 | 65437704 | 1973056 | 88.11% |
| E85-1 | 91843758 | 79379056 | 71296326 | 77474452 | 1904604 | 86.43% |
| E85-2 | 106150115 | 91848112 | 83910954 | 89130130 | 2717982 | 86.53% |
| E85-3 | 102042966 | 88456926 | 80517182 | 85934554 | 2522372 | 86.69% |
| E105-1 | 71794149 | 61773445 | 55774542 | 60029983 | 1743462 | 86.04% |
| E105-2 | 72203100 | 61263463 | 54873234 | 59184711 | 2078752 | 84.85% |
| E105-3 | 70882597 | 59432111 | 53065738 | 57662593 | 1769518 | 83.85% |
| E135-1 | 71897885 | 60345972 | 53804544 | 58197620 | 2148352 | 83.93% |
| E135-2 | 109653263 | 89587473 | 80025624 | 86369479 | 3217994 | 81.70% |
| E135-3 | 90825652 | 75753421 | 68161678 | 73217165 | 2536256 | 83.41% |
| D7-1 | 134267230 | 113448644 | 102694098 | 109725890 | 3722754 | 84.49% |
| D7-2 | 207233829 | 175392658 | 157981912 | 169604088 | 5788570 | 84.64% |
| D7-3 | 68329745 | 58229717 | 52033698 | 56700421 | 1529296 | 85.22% |
| D30-1 | 114326472 | 97114603 | 87447240 | 93986157 | 3128446 | 84.94% |
| D30-2 | 96037526 | 77845262 | 70196342 | 74674298 | 3170964 | 81.06% |
| D30-3 | 84306190 | 70036375 | 62781474 | 67906989 | 2129386 | 83.07% |

Mapping ratio=Mapped reads/All reads; Mapped Unique reads:only one position matching reads in the genome

**Supplementary Table S3. Primers used in qRT-PCR analysis.**

| **Gene name/ID** | **Primer** | **Primer Sequences (5＇－3＇)** | **Product length** | **TM (℃)** |
| --- | --- | --- | --- | --- |
| TCONS_00043783 | F | GGAGGACTGGGCTACAGCTATG | 112 | 60 |
|  | R | AAAATCCAGAGGACAAGTATCTTCCA |  |  |
| TCONS_00091064 | F | TAGGTAGAGAAATGGGGGTTGA | 100 | 60 |
|  | R | CCGCACCTTCAGGACCTC |  |  |
| TCONS_00175219 | F | TTCAGACATTTGGTGTATGTGCT | 100 | 60 |
|  | R | CTGGGCGGGATTCTGACTTA |  |  |
| TCONS_00009946 | F | TCTTTACTCCTTCTTTTTGCCAGC | 79 | 60 |
|  | R | GGAACGCAAGAACAGAATGAAGA |  |  |
| TCONS_00202353 | F | TGACTCTGGCTTGATTTTTTTGG | 75 | 60 |
|  | R | TGAAGCTGTTTGCACGTTACG |  |  |
| TCONS_00268319 | F | ATAGGATTCGCAAGGATGGA | 104 | 60 |
|  | R | AACCCATACCAGTACCATAGACG |  |  |
| TCONS_00298436 | F | AAGTATTCACTGTTGGAGGGAGATG | 85 | 60 |
|  | R | CATTGTTTTATTCCCTAGCCTATTATTTC |  |  |
| TCONS_00280360 | F | TGCTCATGTCTGTCCTTTGG | 101 | 60 |
|  | R | GACTAGTCAACAACCGCCTCA |  |  |
| TCONS_00175038 | F | GGTTGCTGAGTGTGGGTTGA | 103 | 60 |
|  | R | CAAGCCACAAGGTTAAAAGTAAACTG |  |  |
| DKK1 | F | CCAGCGTTGTTACTGTGGAGAAGG | 86 | 60 |
|  | R | AGGTGTGAAGCCTGGAAGAATTGC |  |  |
| SFRP2 | F | gctgtgccacggcatagagt | 73 | 60 |
|  | R | ccgtgccgacctaccagagc |  |  |
| Hoxc13 | F | GCTGCCGCCTGTCTCACAAC | 82 | 60 |
|  | R | ACCGACACGTCCAGGTAGCC |  |  |
| Wnt16 | F | gggcaccagc cctctctttg | 117 | 60 |
|  | R | gcctcgtagggggggttacg |  |  |
| Ovis-actin | F | GCACCCAGCACGATGAAGAT | 102 | 60 |
|  | R | CGCCAATCCACACGGAGTAC |  |  |

F: forward primer; R: revers primer

**Supplementary Figures**


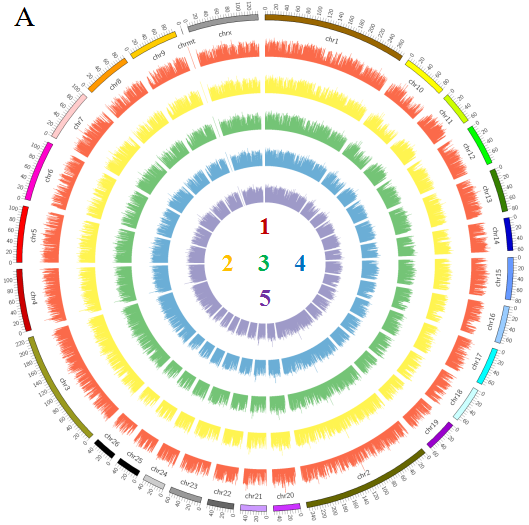

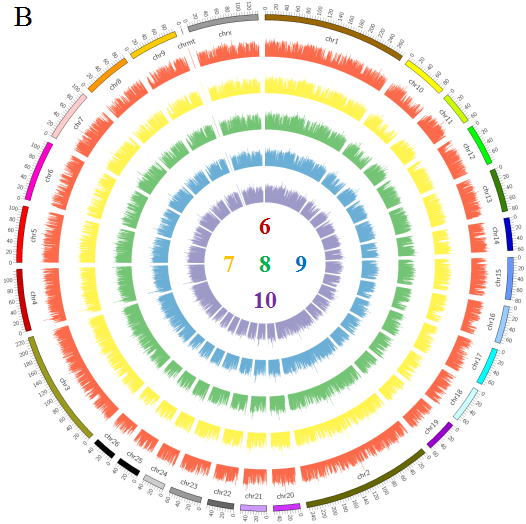


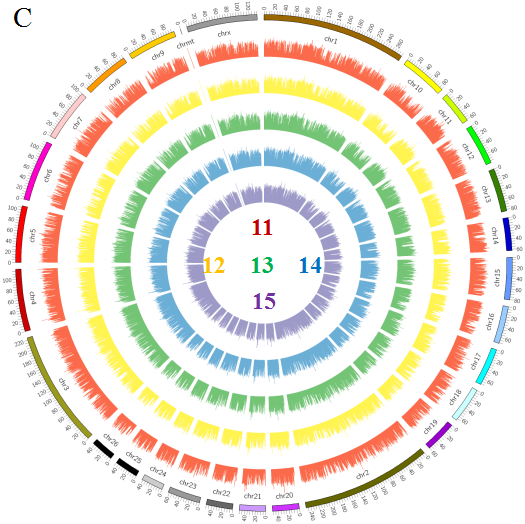

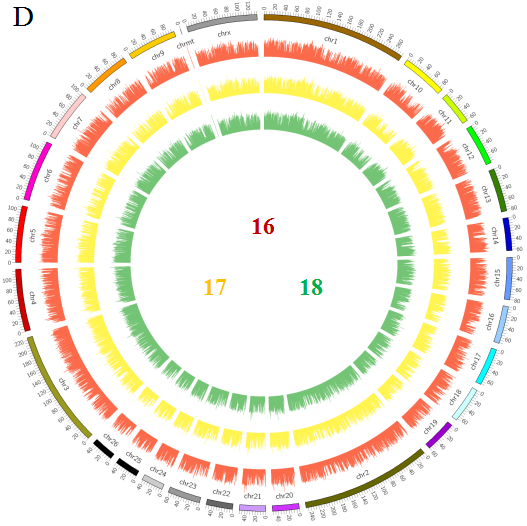


**Supplementary Fig. S1. The distribution map of genome coverage on sheep chromosome.** An overlay distribution of the genome is derived from the 1K window. Genomes are shown in different colors in the outermost circle, and the each innermost circles show the distribution of chromosome coverage of each samples. **A** represents the distribution of genome coverage in sample E65-1, E65-2, E65-3, E85-1, and E85-2. **B** represents the distribution of genome coverage in sample E85-3, E105-1, E105-2, E105-3, and E135-1. **C** represents the distribution of genome coverage in sample E135-2, E135-3, D7-1, D7-2, and D7-3. **D** represents the distribution of genome coverage in sample D30-1, D30-2, and D30-3.


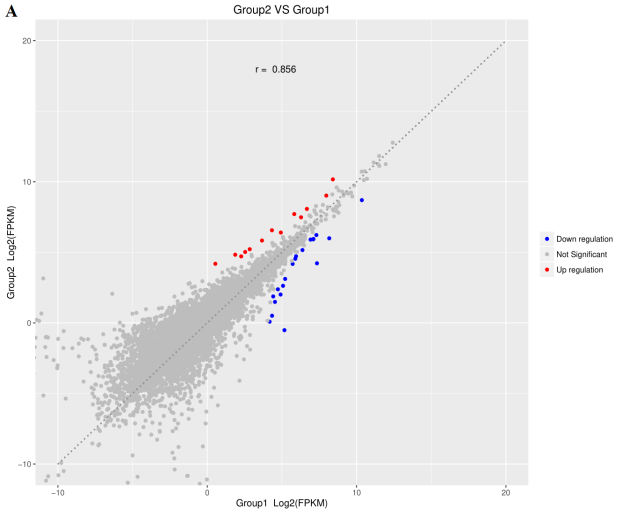

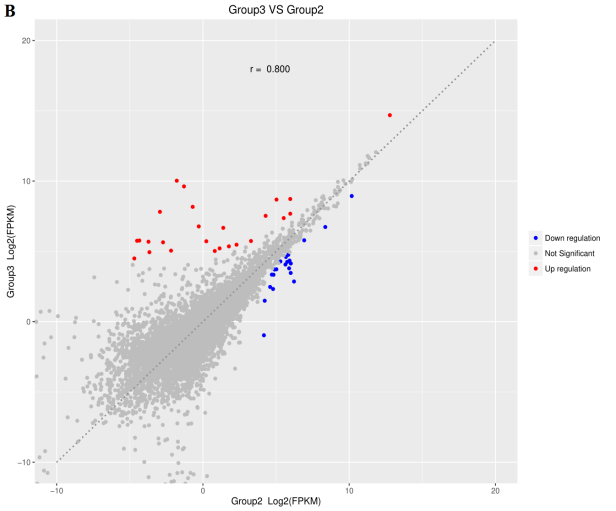


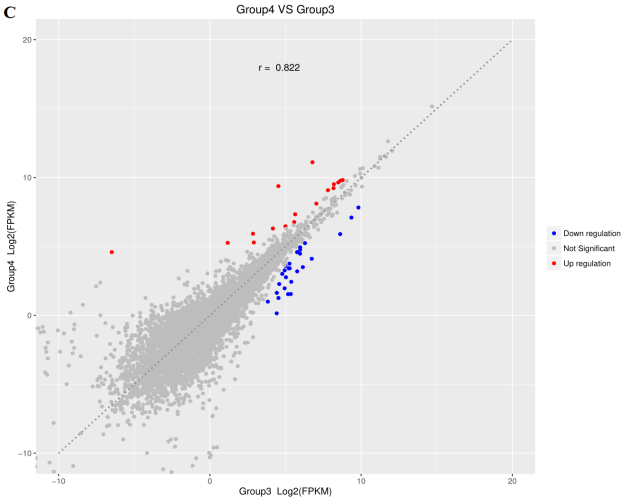

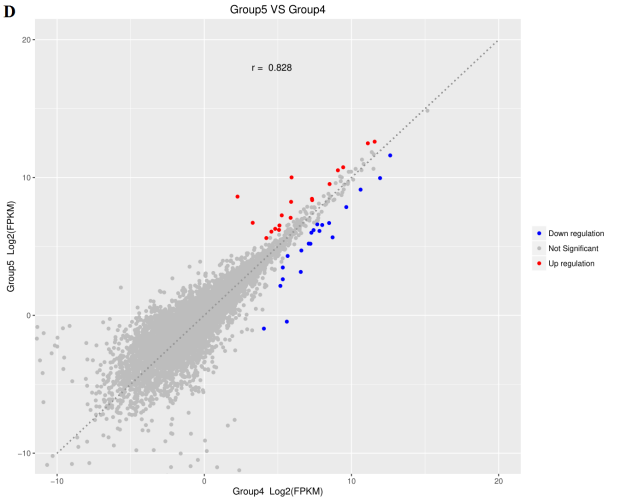


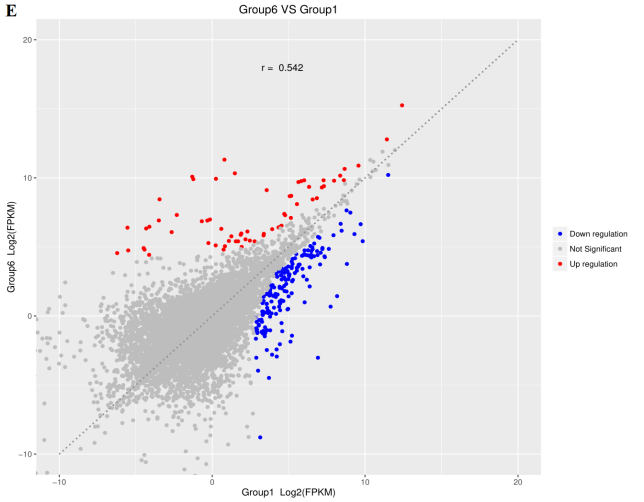

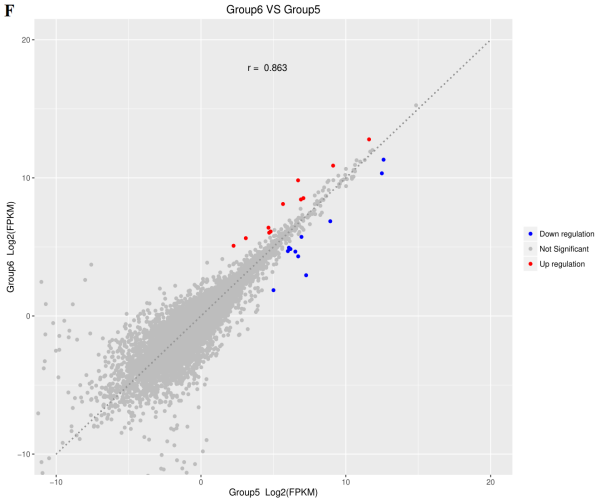


**Supplementary Fig. S2. The differential expressions of sheep skin lncRNAs between different comparison groups are shown.** Each point represents an lncRNA. The X and Y axes show the expression level of lncRNAs in every two samples, respectively. Red point represents the up regulated lncRNAs, and blue point represents the down regulated lncRNAs. **A**: E85-VS-E65; **B**: E105-VS-E85; **C**: E135-VS-E105; **D**: D7-VS-E135; **E**: D30-VS-D7; **F**: D30-VS-E65.


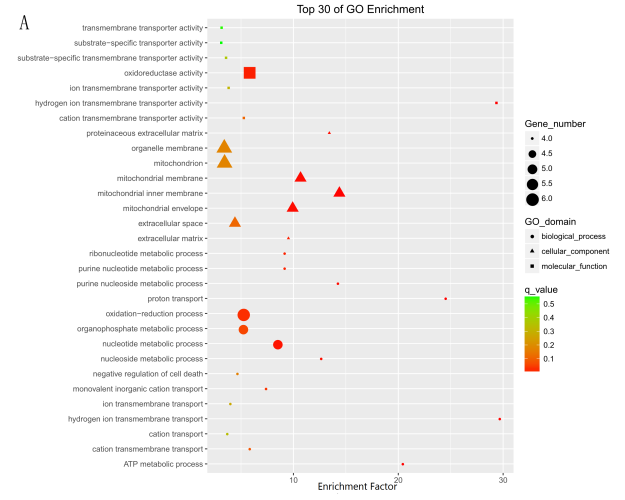

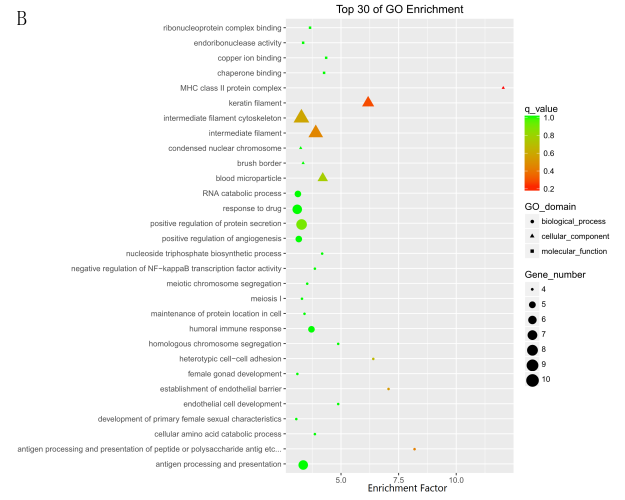


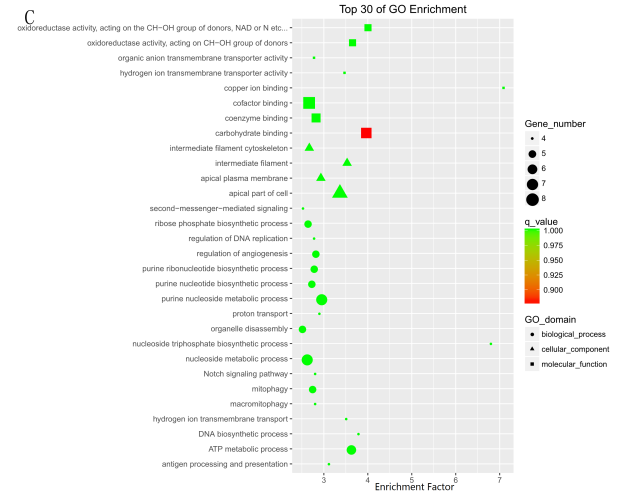

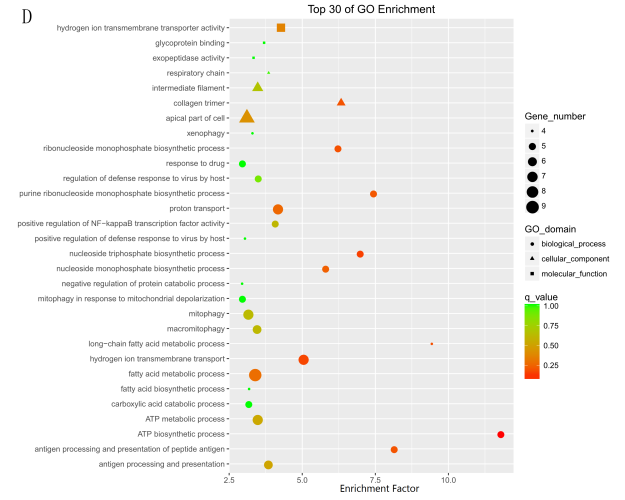


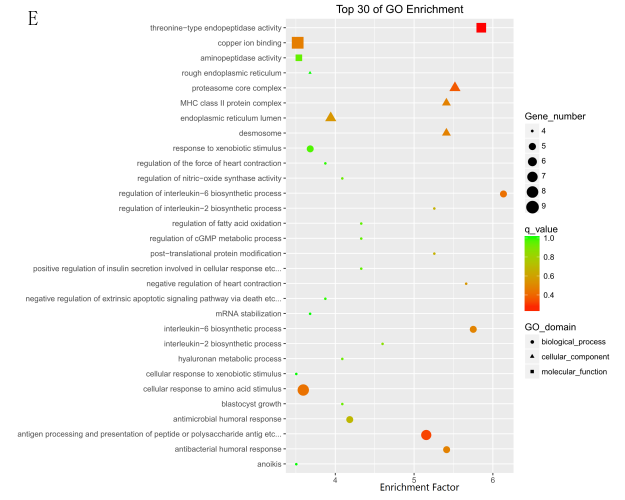

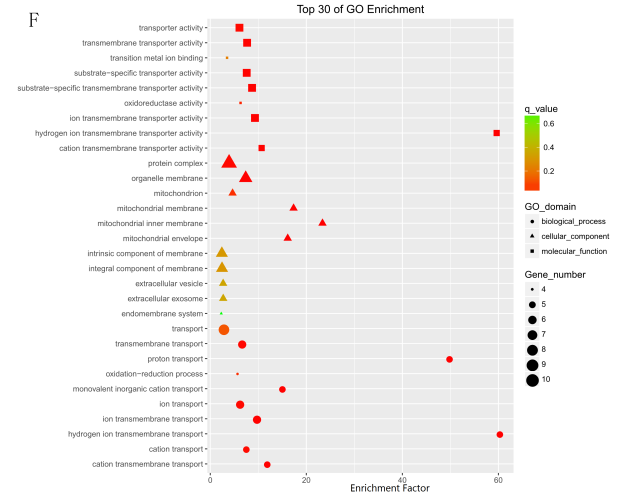


**Supplementary Fig. S3. The top 30 GO enrichment of target genes of differentially expressed lncRNAs in six comparison groups.** The enrichment factor was calculated using the gene count divided by the expected gene count. **A**, **B**, **C**, **D**, **E**, and **F** represent the GO enrichment analysis of target genes of differentially expressed lncRNAs in E85-VS-E65, E105-VS-E85, E135-VS-E105, D7-VS-E135, D30-VS-D7, and D30-VS-E65 comparisons, respectively.


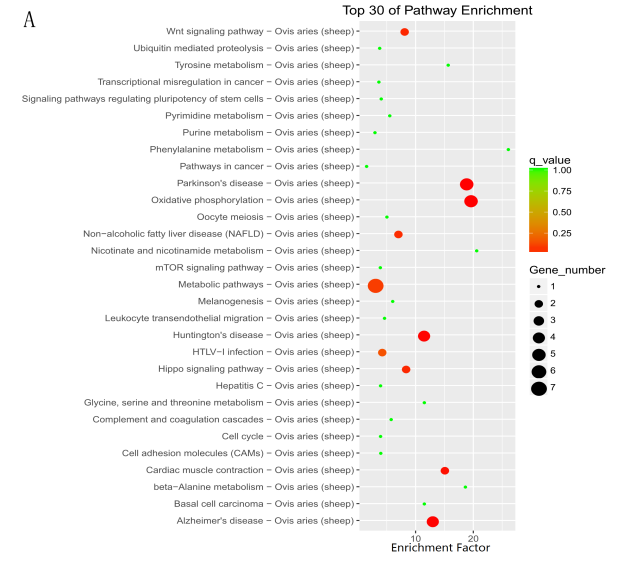

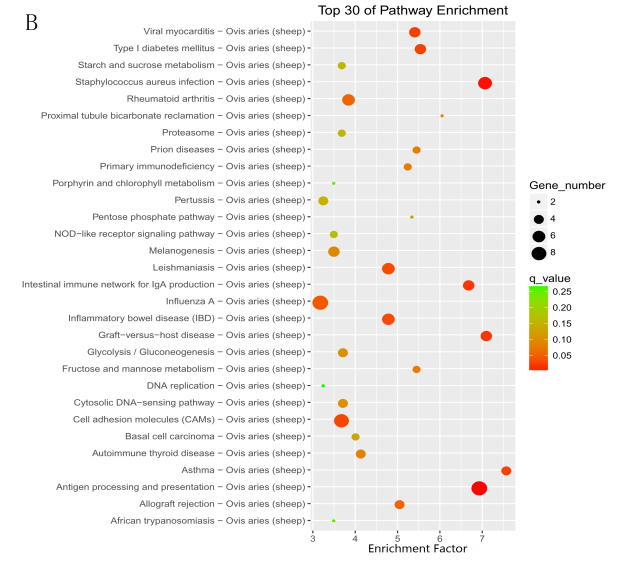


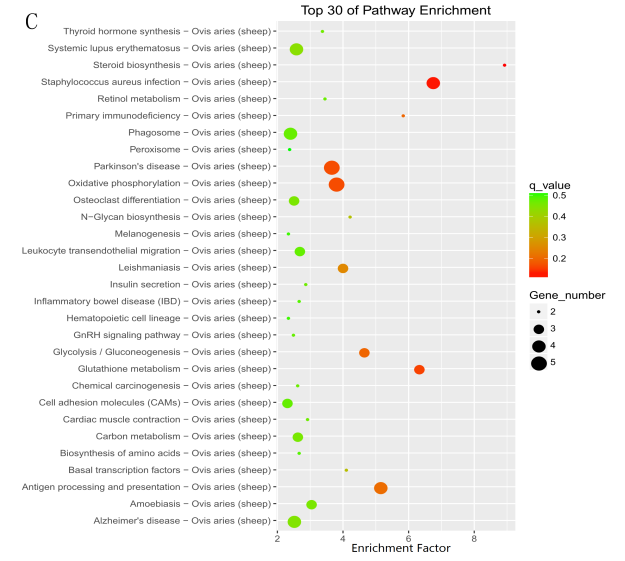

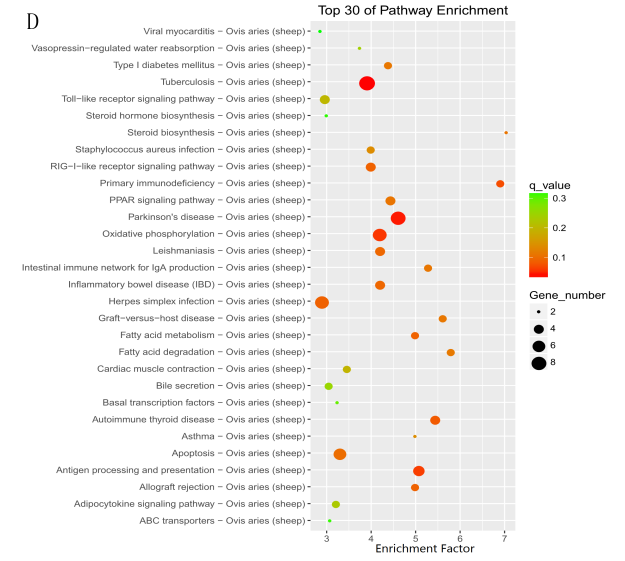


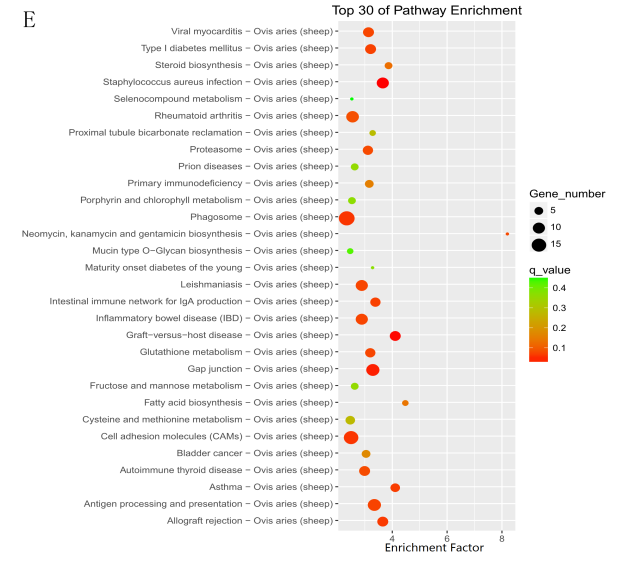

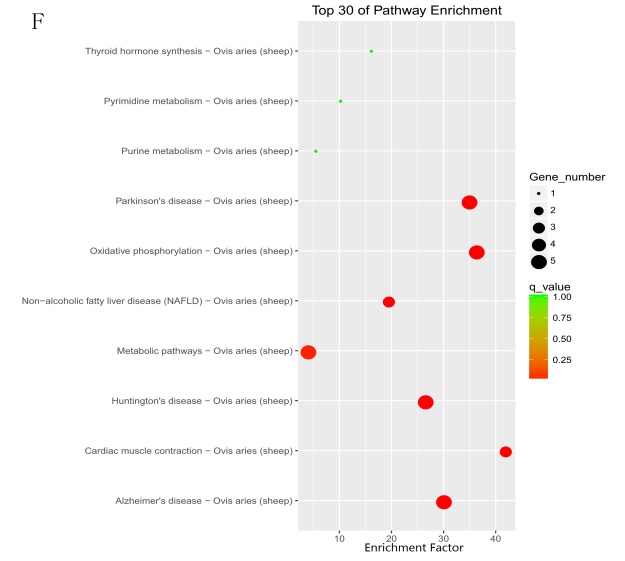


**Supplementary Fig. S4. The top 30 KEGG pathway enrichment of target genes of differentially expressed lncRNAs in six comparison groups.** The enrichment factor was calculated using the gene count divided by the expected gene count. **A**, **B**, **C**, **D**, **E**, and **F** represent the KEGG pathway enrichment analysis of target genes of differentially expressed lncRNAs in E85-VS-E65, E105-VS-E85, E135-VS-E105, D7-VS-E135, D30-VS-D7, and D30-VS-E65 comparisons, respectively.
